# Community-acquired infection with hypervirulent *Clostridium difficile* isolates that carry different toxin and antibiotic resistance loci: a case report

**DOI:** 10.1186/s13099-017-0212-y

**Published:** 2017-11-09

**Authors:** Marina Muñoz, Milena Camargo, Dora Inés Ríos-Chaparro, Paula Gómez, Manuel Alfonso Patarroyo, Juan David Ramírez

**Affiliations:** 1 Universidad del Rosario, Facultad de Ciencias Naturales y Matemáticas, Programa de Biología, Grupo de Investigaciones Microbiológicas–UR (GIMUR), Bogotá, Colombia; 2 Posgrado Interfacultades Doctorado en Biotecnología, Facultad de Ciencias, Universidad Nacional de Colombia, Bogotá, Colombia; 3 Molecular Biology and Immunology Department, Fundación Instituto de Inmunología de Colombia (FIDIC), Bogotá, Colombia; 4 Universidad del Rosario, School of Medicine and Health Sciences, Bogotá, Colombia

**Keywords:** *Clostridium difficile*, Toxigenic profiles, Antibiotic resistance, Hypervirulent strain

## Abstract

**Background:**

*Clostridium difficile* infection (CDI) leads to the onset of antibiotic-associated diarrhea (AAD) and a wide range of gastrointestinal pathologies. Currently, CDI is one of the most important opportunistic infections at the intrahospital level and an exponential increase in community-acquired infections has been reported. Herein, we evaluated the relationships (at phylogenetic and genetic population structure levels), as well as the molecular toxigenic and antibiotic resistance profiles of a set of isolates established from a case of community acquired-CDI.

**Case presentation:**

A 30-year-old woman with no history of hospitalization who was exposed to antibiotics (ampicillin/sulbactam and metronidazole) after a cat-bite wound was presented. The patient had a continuous episode of diarrhea; a stool sample was then collected and community acquired-CDI was confirmed by molecular tests and in vitro culture. Seven isolates were established and subsequently subjected to: (i) Multilocus sequence typing, all isolates belonging to ST-1 (associated with hypervirulent strain (027/BI/NAP1); (ii) description of their toxigenic profile: two of the isolates (Gcol.49 and Gcol.91) were positive for the genes coding for the major toxins (*tcdA* and *tcdB*) and their negative regulator (*tcdC*). All isolates were positive for the *cdtB* gene encoding one of the binary toxin subunits, while only two (Gcol.51 and Gcol.52) were positive for *cdtA*; and (iii) identification of antibiotic resistance molecular markers, where there was no difference in *gyrA* or *gyrB* gene polymorphisms (related to quinolone resistance), but rather at loci presence/absence, being just one isolate negative, whereas the others showed a differential presence of the *tet*, *ermB* and Tn916 regions. The former was associated with resistance to tetracycline and the other two for erythromycin/clindamycin.

**Conclusions:**

This case represents the first report of community acquired-CDI in Colombia associated with hypervirulent strains and shows that isolates obtained from a single patient can carry different toxin and antibiotic resistance loci.

**Electronic supplementary material:**

The online version of this article (10.1186/s13099-017-0212-y) contains supplementary material, which is available to authorized users.

## Background

*Clostridium difficile* (CD) may be present at the gastrointestinal level as a commensal in a small group of healthy adult individuals [1]. Its proliferation triggers a wide range of pathologies defined together as CD infection (CDI), ranging from antibiotic-associated diarrhea (AAD) to pseudomembranous colitis, toxic megacolon and even death [2]. The globally accepted typing strategy for CD is multilocus sequence typing (MLST), where allelic variation of housekeeping genes is identified, and each particular allelic profile allows to assign a sequence type (ST). A globally accepted MLST scheme for CD typing is available [3] within the PubMLST databases (CD-MLST-db; https://pubmlst.org/cdifficile/), providing the most robust MLST data collection, where, in addition to identifying the allelic profiles of query sequences, the sequences of both, the MLST scheme and other clinically important loci are publicly available. This data repository has been the basis for assessing intra-taxa diversity (identifying 6 clades (C), denominated C1 to C5, and a highly diverse additional clade, proposed as C-I) [4].

The main virulence factors of CD are TcdA and TcdB toxins, encoded by the *tcdA* and *tcdB* genes, respectively, which belong to the clostridial toxin family and display glucosyltransferase action [5]. These toxins are primarily responsible for the presentation of symptoms [6] and are encoded by genes located in a chromosomal region called the locus of pathogenicity (*PaLoc*) [7]. Polymorphisms have been identified across the *PaLoc*, related to various degrees of CD virulence. The most obvious case is the hypervirulent ribotype 027 (BI/NAP1), which belongs to ST-1 (C2) and has a deletion of one base pair at position 117 of the *tcdC* gene. Some isolates have the ability to produce binary toxin, which has ADP-ribosyl transferase activity and is encoded by a chromosome region called *CdtLoc*, containing the *cdtA* and *cdtB* genes, coding for the two subunits of this toxin and a transcriptional regulator (*cdtR*) [8].

The acquisition of antibiotic resistance in CD has been associated with the existence of molecular markers that in some cases may correspond to mutations in genes, such as *gyrA* and *gyrB*, and associated with resistance to fluoroquinolones [9]. However, in most cases, there are modules that confer resistance to antibiotics, such as *erm* (B) genes (conferring resistance to erythromycin and clindamycin) [10, 11] and the *tet* (M) and *tet* (W) genes (related to tetracycline resistance) [12, 13].

The increase in the impact of CDI reported during the last 15 years has been attributed to the plasticity of its genome. Due to the mobilization of loci related to the production of toxins and the acquisition of antibiotic resistance, favoring the emergence and dispersion of hypervirulent strains [14]. These features could cause clinical manifestations of greater impact (mainly in strains producing the three toxins) [15] and by having alarming incidence and mortality rates, causing outbreaks on different countries [16, 17]. This differential impact of certain CD populations highlights the need to carry out a toxigenic characterization to identify the circulation of those strains having clinical and epidemiological importance [18]. These infection patterns have been widely described in developed countries, however, they have just been evaluated at intra-hospital level in different countries [19], and the impact of community-acquired CDI remains unknown in such regions.

Therefore, the molecular characterization of isolates obtained from a community-acquired CDI was carried out to determine the ST and clade via MLST, to evaluate phylogenetic relationships and population genetic structure, by comparison with STs reported in the CD-MLST-db. Additionally, toxigenic profiles of these isolates and antibiotic resistance molecular markers were determined as valuable tools to predict their potential impact on the host.

## Case presentation

A 30-year-old woman was admitted at the emergency room with a wound in her upper right limb after a cat bite. Physical examination showed good general conditions with a 2 cm diameter wound, accompanied by pain and purulent discharge. As part of the management scheme, ampicillin/sulbactam was formulated. After 5 days of antibiotic treatment, the patient reported diarrhea which persisted for a week. The patient returned to a consultation where the clinical picture was attributed to a parasitic infection, and was subsequently treated with metronidazole for 7 days. The diarrhea worsened presenting abundant feces with mucus and macroscopic blood. Coproscopic examination was performed with the following results: negative for intestinal parasites, pH: 5.0, leukocytes: positive, 40–50 red blood cells per field, reducing sugars: negative. The patient remained with diarrhea for 20 days, with mucus and blood. Therefore, it was suspected that it could be related to the use of antibiotics. Then, a stool sample was collected to evaluate the presence of CDI.

The stool sample was subjected to two CDI detection methods, following the methodology implemented in a previous study by our group (Munoz et al. unpublished data). The first strategy was via molecular diagnosis, for which an aliquot of 300 μL of the stool sample was subjected to DNA extraction using the Stool DNA Isolation Kit (Norgen Biotek Corporation, Ontario, Canada), according to the manufacturer’s recommendations. An aliquot of 30 ng of the extracted DNA was used to conduct two conventional polymerase chain reaction (PCR) tests: one targeting *16S ribosomal RNA* [20] and the other targeting gene encodes Glutamate dehydrogenase enzyme *(gdh)* [21]. The second strategy to detect CDI was through in vitro culture; for this purpose, an initial portion of the diarrheic stool specimen was extended by streaking method on selective chromogenic medium [(SCM; chromID *C. difficile* agar CDIF (bioMérieux SA, Craponne, France)], followed by 48 h of incubation at 37 °C under anaerobic conditions. The two tests implemented were positive whereby was defined as CDI. Because the patient had not been hospitalized during the 12 weeks prior to the presentation of diarrhea, she was defined as a community-acquired CDI [22].

All colonies identified on SCM were subjected to verification by colony screening through their recovery on Trypticase ™ I Agar (TSA) with 5% Sheep Blood (Becton–Dickinson, New Jersey, United States), followed by confirmation of their microscopic morphology by Gram staining (gram positive bacillus, occasionally sporulated). A total of seven colonies were verified and subsequently used for establishing isolates, increasing the biomass on TSA, followed by recovery for two purposes: (i) cryopreservation through recovery in 500 μL of Oxoid Nutrient Broth (Thermo Fisher Scientific, Massachusetts, United States) with 20% (v/v) Glycerol (Thermo Fisher Scientific) until reaching an optical density (OD 600) of 4 × 10^7^ cells per mL and subsequent storage at − 80 °C, and ii) as a source of DNA for molecular analyses, by recovering a similar amount of cells in 300 μL sterile phosphate buffered saline (PBS) 1×, which was then subjected to extraction using the commercial UltraClean^®^ BloodSpin^®^ DNA Isolation Kit (MoBio Laboratories, Carlsbad, United States), following the manufacturer’s instructions. All isolates established for this patient were analyzed, due to the possible coexistence of different CD genotypes [23].

**Fig. 1:**
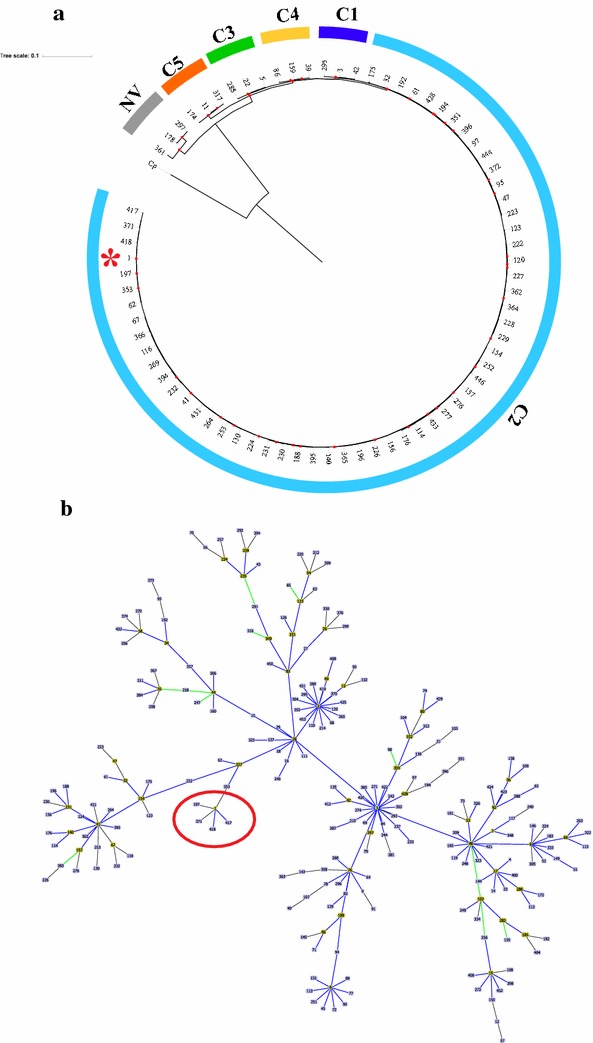
Phylogenetic analysis and population genetic structure of Sequences Type (ST) 1 and related STs. **a** Maximum-likelihood tree based on the concatenated alignment of the seven housekeeping genes used in the MLST scheme. For phylogenetic reconstructions, sequences of the total STs reported for the clade (associated with hypervirulent strains) and representative STs of the other CD clades (selected under the criteria described in Additional file 1) were used. The red points represent Bootstraps > 80%. The ST-1 (isolates from this study) has been marked with a red asterisk. **b** Population genetic structure of clonal complex 1, based on the eBURST algorithm. Each box represents the Sequence Type, with the founder STs marked in yellow. A red circle was used to mark the node where the ST-1 is a founder
(ii) Determining the toxigenic profile through the identification of *PaLoc* and *CdtLoc* regions: The scheme for describing the toxigenic profile proposed by Griffiths et al. [3] was implemented, which targets the identification of genes encoding the major toxins (*tcdA* and *tcdB*), and their negative regulator (*tcdC*). The results showed that only two of the isolates (Gcol.49 and Gcol.91) were positive for the *tcdA* and *tcdB* genes, and the negative *tcdC* regulator (Fig. 2a). In addition, this scheme includes the set of lok1/3 primers, that anneal in the cdd1/cdu1 genes flanking the *PaLoc* (as an indicator of *PaLoc* absence). Interestingly, in the case of Gcol.49, a positive PCR result was also identified with the lok1/3 primers, although their amplification size was ~ 300 base pairs (bp), less than half the expected size (769 bp). The presence of an amplicon of this size was considered positive as this was confirmed by Sanger sequencing corresponding to CD (Additional file 2). In addition to the toxigenic profile description, primers targeting genes encoding the binary toxin (*cdtA*/*cdtB*) subunits, located within the *CdtLoc* were used [26]. We found that all isolates were positive for the *cdtB* region, whereas only two (Gcol.51 and Gcol.52) were positive for *cdtA* (Fig. 2b).

From the DNA extracted from the seven isolates, the following molecular tests and analyzes were performed:

(i) MLST typing: Regions of the seven housekeeping genes included in the MLST scheme proposed by Griffiths et al. [3], were amplified independently and sequenced by the Sanger method. The sequences obtained for each isolate were compared against the previously reported profiles, using the ‘locus/sequence definitions database’ tool available on the MLST database (CD-MLST-db; https://pubmlst.org/bigsdb?db=pubmlst_cdifficile_seqdef). The results indicated that all isolates belonged to the ST-1 (clade 2), which has previously been associated with the hypervirulent strain 027/BI/NAP1 [14]. Phylogenetic relationships of ST-1 with other STs belonging to C2 (n: 60) and 3 STs representatives from each other clades (included for comparative purposes), were evaluated. Then, the concatenated sequences of the seven housekeeping genes of the STs reported on CD-MLST-db were aligned using multiple sequence comparison by log-expectation (MUSCLE) [24] and later used for the inference of phylogenetic reconstructions using the maximum-likelihood method, considering Jukes-Cantor as the model of nucleotide evolution. Node robustness was evaluated using the Bootstrap (BT) method with 1000 replicates. A cluster was defined from the nodes with BT results > 80%. The graph visualization of the phylogenetic trees was done in the web-based tool Interactive Tree Of Life V3 (http://itol.embl.de) [25]. The concatenated sequences of homologous genes in *Clostridium perfringens* were included as outgroup. The results showed that the concatenated sequences of the seven housekeeping genes allowed clade discrimination, in agreement with previous reports [4, 15]. In the case of C2, it could be identified that 60 STs are grouped in 30 clusters (BT ≥ 80), and that it is closely related to C1 (Fig. 1a) [4]. The selection criteria of the representatives of the other clades and the information of the complete set of STs used in phylogenetic reconstructions is described in Additional file 1. In addition, analysis of the population genetic structure based on the application of the eBURST algorithm on the total of STs reported for CD, allowed us to confirm that this ST belongs to the clonal complex 1 (CC1), which includes 245 STs of the 454 reported according with the last update of the CD-MLST-db sequence database (2017-09-29). For this CC1, the predicted founding genotype is ST-3, and in the case of ST-1 it is identified as a founding subgroup for STs: 197, 371, 417 and 418 (Fig. 1b).

**Fig. 2:**
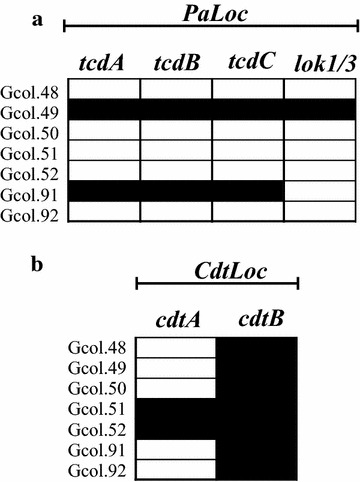
Amplification profiles of the coding regions for the toxins. **a** Amplification profiles of encoding regions for the major clostridial toxins, located within the *PaLoc*. **b** Amplification profiles of coding regions for the binary toxin subunits, located within the *CdtLoc*. Black rectangles represent a positive result for each test, while white rectangles a negative result
(iii) Identification of molecular markers of antibiotic resistance: An initial approach was aimed at identifying polymorphisms in the *gyrA* and *gyrB* genes, determinants of quinolone resistance. Then, regions of such genes were amplified and sequenced following a previously proposed methodology [9]. The sequences obtained were compared with CD-MLST-db, with all isolates corresponding to the 65 alleles for *gyrA* and 50 for *gyrB*. Phylogenetic reconstructions were obtained from the complete set of alleles reported for each gene (118 and 98 for *gryA* and *gyrB*, respectively), under the parameters previously described. This approach identified 18 clades for *gyrA* (Fig. 3a), while 16 were identified for *gyrB* (Fig. 3b). These findings could be related to the high identity of these housekeeping genes. A second approach at the level of antibiotic resistance markers was aimed at evaluating the presence/absence of cassettes, through the amplification of: *ermB*, related to resistance to erythromycin and clindamycin, *tetM*, conferring resistance to tetracycline, and the *tndX* and *Int* regions as indicators of the presence of Tn5397 and Tn916-like elements. The results showed that there is no circulation of Tn5397 type elements in these isolates. However, the other markers were found in all isolates except Gcol.52. The other isolates showed different profiles of positivity for the markers. These are described in Fig. 3c. DNA extracted from the strains: ATCC BAA-1870 (*tcdA*, *tcdB* and *cdt* presence confirmed by PCR) and ATCC 700057 (toxinotype *tcdA*-, *tcdB*- and binary toxin gene *cdtB* not amplified by PCR) was included as controls for all tests implemented. The information of the primers employed for the different molecular tests is described in Additional file 3.

**Fig. 3:**
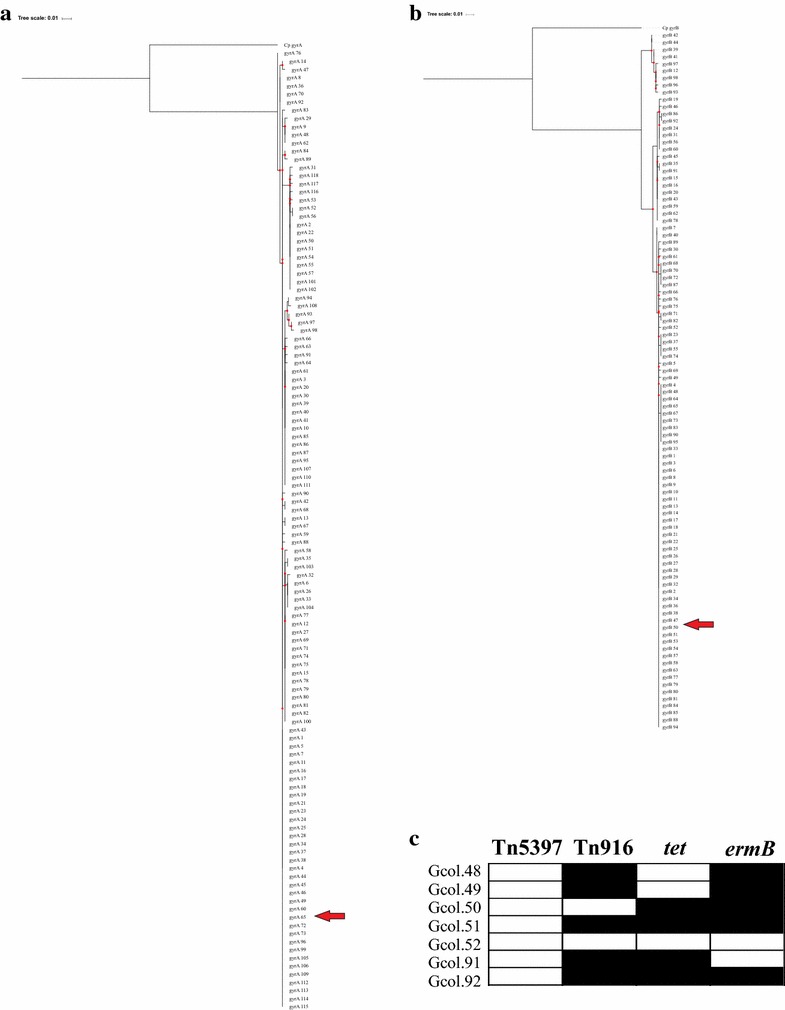
Evaluation of antibiotic resistance markers **a** and **b** Phylogenetic reconstructions inferred by the maximum-likelihood method from alignments of the alleles reported for the *gyrA* and *gyrB* genes, respectively, associated with quinolone resistance. **c** Amplification profiles of loci associated with resistance to erythromycin and clindamycin (*ermB*) and tetracycline (*tet*), as well as detection of Tn5397 and Tn916-like elements. In **a** and **b**, the red arrows show the allele that carry the isolates for each of the genes. In **c**, the black rectangles represent a positive result for each test, while white rectangles a negative result

Finally, after the identification and characterization of the CDI, nutritional management was performed with food intake rich in probiotics. This strategy led to the improvement of the clinical picture of the patient.

## Discussion and conclusions

CDI has historically been considered relevant at the nosocomial level, despite the increasing cases of CDI acquired in the community. However, most studies analyze samples from hospital sources [19], leading to an underestimation of the prevalence and impact of CDI. Although its presence does not generate a relevant impact on the health conditions of immunocompetent individuals, its potential proliferation under dysbiotic conditions may become a route of CDI dissemination [22]. In this study, the prolonged diarrhea was of particular interest considering that it occurred in a 30-year-old woman with no history of hospitalization or other concomitant diseases. Thus, the only identifiable risk factor was exposure to antibiotics, which is in line with the widely reported information for CDI [27]. These findings show once again the remarkable importance of the adequate use of antibiotics within the framework of the management of concomitant diseases, even in individuals of the community.

The association of the isolates obtained with the hypervirulent strain (ST-1) and other STs related to epidemiological outbreaks belonging to the same clade (C2; Fig. 1a, b), allows us to propose a broader epidemiological profile for these strains, where they could be present in asymptomatic carriers [22]. Although studies directed to detect CDI associated with hypervirulent strains have been previously reported in some Latin-American countries (including Colombia). All these reports correspond to infections acquired at intra-hospital level [19, 28]. Therefore, these findings represent to our knowledge the first robust characterization of toxigenic profiles and molecular resistance markers of a community-acquired CDI in the region (Latin America).

During the characterization process of the CDI, the detection of molecular markers located within the *PaLoc* represent an effective tool to monitor the potential cytotoxic effect of colonizing strains [29]. However, a high level of variation reported has been related with false-negative results when diagnostic tests are implemented for these genes [30]. This strategy was then directed to the characterization of isolates, revealing different *PaLoc* organizations within CD clades [31] and sequence variation in epidemic strains [32], which agrees with the results identified for this set of isolates (Fig. 2). However, there are reports of dispersion of the hypervirulent strains across Latin America [33], even including a description of regions of *PaLoc* and *CdtLoc* [34]. These correspond exclusively to hospital-acquired CDI.

Regarding the molecular markers associated with antibiotic resistance, no difference was found between mutations in the *gyrA* and *gyrB* genes (associated with resistance to fluoroquinolones) [9] (Fig. 3a and b, respectively). On the other hand, a differential presence of loci associated with resistance was identified, as is the case for *ermB* (conferring resistance to erythromycin and clindamycin) [11] and *tet* (related to tetracycline resistance) [12]. In addition, these loci were present simultaneously in the same isolate (in Gcol.50, 51 and 92, Fib 3C), which agrees with previous reports where heteroresistance to antibiotics has been identified in isolates associated with epidemic strains [35]. The differential presence of the Tn916 loci (marker of transposable elements of conjugative type) [11], could be an indicator of the pathway of acquisition of these loci. Together, these data are consistent with previously reports in CD, where molecular profiles of multidrug-resistance have been associated with mobile genetic elements, and in particular with conjugative transposons [36].

In general, the differential identification of the molecular markers evaluated (associated with both toxins and antibiotic resistance) in these isolates, confirm the importance of evaluating different isolates from a single patient to accurately determining the toxigenic potential and antibiotic resistance associated with CDI. This can be miss-determined by conventional diagnostic methods that can lead to prolonged use of antibiotics or inadequate treatment of these infections. Although in this case, it was identified that the isolates corresponded to a single ST, the differential presence of these molecular markers confirms the high frequency of genetic material exchange of CD, which can occur either by horizontal gene transfer or by the presence of mobile genetic elements [37]. These findings represent a new indicator of the genome plasticity of CD [14] and is consistent with previously described host adaptation strategies for the epidemic strain 027/BI/NAP1 [38]. However, further characterization through whole genome sequencing of the isolates is required to clarify the molecular basis of these variations.

These findings suggest that the implementation of molecular tests aimed at characterizing housekeeping genes (MLST) does not represent the best strategy to describe the molecular epidemiology of CD or to monitor epidemiological outbreaks. In the future, it is necessary to design strategies that allow the detection of clinically relevant loci, such as genes encoding for toxins or loci that confer resistance to antibiotics to predict the potential impact of the infective strain on the host. It is important to consider that in developing countries such as Colombia, where a regulated CDI diagnosis scheme is not yet available, the timely detection of this type of organization at the molecular level could represent a practical tool to improve management of CDI positive patients. The clinical impact of nucleic acid amplification tests is significant, since they are much more sensitive and specific, allowing the identification of the biological characteristics of the infecting strain, in addition to the generation of reliable results in short periods of time [1].

## Conclusions

In conclusion, these findings allowed us to identify a community-acquired CDI for the first time in Colombia and additionally demonstrate that the CD present in the same individual can carry different toxin and antibiotic resistance related loci in spite of belonging to the same ST. This evidence contributes new light on the virulence factors of this species and can represent a source of information for the improvement of the management strategies of the patient at therapeutic level and for the implementation of measures for the control of CDI from non-hospital sources.

## Additional files

**Additional file 1.** Selection criteria for representatives of other clades and complete set information of STs used in phylogenetic reconstructions based on the concatenated sequence of the seven housekeeping genes used in the MLST scheme.

**Additional file 2.**BLAST results of the sanger sequencing using primers lok1/3.

**Additional file 3.**Information of the primers employed for the different molecular tests conducted.

## Abbreviations

AAD: antibiotic-associated diarrhea
C: clade
CD: *Clostridium difficile*
CDI: *Clostridium difficile* infection
CD-MLST-db: *Clostridium difficile* multilocus sequence typing database
MLST: multilocus sequence typing
ST: sequence type
