# Supplementary material for: Community-acquired infection with hypervirulent Clostridium difficile isolates that carry different toxin and antibiotic resistance loci: a case report
Source: Gut Pathog. 2017 Nov 9;9:63. doi: 10.1186/s13099-017-0212-y (PMC5680771; doi:10.1186/s13099-017-0212-y)
Supplement: Supplementary file 1 — Additional file 1. Selection criteria for representatives of other clades and complete set information of STs used in phylogenetic reconstructions based on the concatenated sequence of the seven housekeeping genes used in the MLST scheme. [file 13099_2017_212_MOESM1_ESM.docx]

**Additional file 1.** Selection criteria for representatives of other clades and complete set information of STs used in phylogenetic reconstructions based on the concatenated sequence of the seven housekeeping genes used in the MLST scheme.

The STs included as representatives of the other clades were selected considering the results of a previous work of our research group, where we evaluated the intra-taxa diversity of CD on the basis of Multilocus sequence typing data of the total STs reported on CD -MLST-db (https://pubmlst.org/cdifficile/) [1]. The selection criteria were: i) to be grouped with the members of the same clade to which it is assigned in the database, in the phylogenetic inferences based on the concatenated sequence used for the proposal of the intra-taxa classification made in previously published paper (1), and ii) They belonged to a cluster differentiated during the analysis of phylogenetic networks based on the algorithm neighbornnet available in the software SplitsTree, carried out in the same study. According to the findings of this previous report, a cluster of isolates that is not assigned to any clade in the CD-MLST-db (NV, No value), was consistently identified as a cluster within the classifications generated by both methods. Then, it was considered as a separate cluster for the selection of representative STs.

The following describes the list of STs belonging to the other clades, selected under these parameters:

| Clade-1 | Clade-3 | Clade-4 | Clade-5 | NV |
| --- | --- | --- | --- | --- |
| 3  42  295 | 5  22  285 | 39  86  159 | 11  174  317 | 178  297  361 |

**Reference**

1. Munoz M, Rios-Chaparro DI, Patarroyo MA, Ramirez JD: **Determining Clostridium difficile intra-taxa diversity by mining multilocus sequence typing databases.** *BMC Microbiol* 2017, **17:**62. doi: 10.1186/s12866-017-0969-7.
